# Preclinical Demonstration of a Novel Treatment with High Efficacy and No Detectable Toxicity for Inflammatory Skin Conditions including Psoriasis

**DOI:** 10.1155/2023/4878774

**Published:** 2023-07-11

**Authors:** John P. Vanden Heuvel, Shuling Zhou, Anisha B. Patel, Harry N. Kamerow, Peter Baran, John P. Ford

**Affiliations:** ^1^Department of Veterinary and Biomedical Sciences, Penn State University, University Park, PA 16802, USA; ^2^Asymmetric Therapeutics LLC, 141 Main St., P.O. Box J, Unadilla, NY 13849, USA; ^3^Indigo Biosciences, Inc., 3006 Research Drive, State College, PA 16801, USA; ^4^Department of Dermatology, Division of Internal Medicine, The University of Texas MD Anderson Cancer Center, Houston, TX, USA; ^5^Mount Nittany Medical Center, 1850 East Park Avenue, State College, PA 16803, USA

## Abstract

Although the management options for psoriasis have progressed with the use of systemic agents, there are few efficacious nonsteroidal topical therapies for patients with limited or lower grade disease. The effects of allopurinol (Allo) and glutathione (GSH) were examined in two different *in vitro* models for psoriasis. In the first model, human immortalized keratinocytes (HaCaT) were treated with M5 cocktail (IL-17A, IL-22, oncostatin M, IL-1*α*, and TNF-*α*) in four interventional groups (control, Allo, oxypurinol (Oxy), and methotrexate (MTX)). The number of live and dead cells was determined after treatment for 48 and 72 hrs. Allo decreased cell proliferation (total cells) without increasing cell death compared to both its xanthine oxidase inhibiting metabolite Oxy and a standard agent in clinical use, MTX. In the second model, a human psoriatic skin equivalent (PSE) culture system, cells were treated with vehicle control, Allo and GSH (as monotherapies and in combination), and vitamin D (VitD) for 2 and 6 days followed by histological analysis and altered gene expression. The combined exposure to Allo and GSH was equivalent to a standard antipsoriasis agent VitD in the inhibition of both proliferative and replicative markers. Histologic examination of the tissue at 6 days of exposure to VitD resulted in loss of the integrity of the squamous/epithelial continuity whereas tissue integrity was preserved with Allo and GSH exposure. The additional exposure of GSH to Allo reversed the increased thickness of the dermis layer caused by Allo exposure alone. Taken together, this data shows that topical Allo and GSH may have a synergistic effect with low toxicity and constitute a therapeutic advantage over current nonsteroidal therapies in the treatment of inflammatory skin conditions marked by increased cell proliferation such as psoriasis.

## 1. Introduction

A common feature of many inflammatory skin conditions is more rapid cell replication; in psoriasis, the cell doubling time is 3-5 days compared to 30 days in the skin of normal individuals [1, 2]. Restoring the slower rate of cell proliferation and preserving normal tissue architecture to approximate that of normal skin could produce a therapeutic benefit like that achieved with the modulation of the immune pathways but with a better therapeutic index.

Many current efforts to control inflammatory skin conditions target cell factors that modulate inflammatory or immune response. The redundancy of these homeostatic networks makes achieving a therapeutic response while obtaining an acceptable therapeutic index a challenge. Such efforts have included anticytokine biologics targeting interleukin-17 (IL17) or its ligand IL17A, IL23, and tumor necrosis factor alpha (TNF-*α*) [3]. The small molecule drug apremilast (marketed under the trade name Otezla® by Amgen Inc., Thousand Oaks, CA), a selective inhibitor of the enzyme phosphodiesterase 4 (PDE4) that inhibits production of TNF-*α* by rheumatoid synovial cells, among others, is increasingly being used to treat psoriasis [4]. Additionally, these immune modulators, at low incidence, carry the risk of serious toxicities, including cancer and activating tuberculosis.

Oral allopurinol (Allo) has been tested in the treatment of psoriasis showing first a benefit (5), but then, there is no difference in a randomized trial [5, 6]. In 1970, Fox et al. noted that Allo, a xanthine oxidase (XO) inhibitor widely used clinically in the treatment of gout, leads to the depletion of phosphoribosyl pyrophosphate (PRPP) in patients [7]. PRPP is a necessary intermediate in the synthesis of both purine and *de novo* pyrimidine ribonucleosides required for both DNA and RNA syntheses. This is not the case for oxypurinol (Oxy), the Allo metabolite that is the direct inhibitor of XO [7]. Allo but not Oxy is a substrate for hypoxanthine phosphoribosyl transferase (HGPRT) and could lead to PRPP depletion [8, 9, 10] and explain the Fox findings. We reasoned that the depletion of PRPP would be expected to be deleterious in the psoriasis model systems and Oxy would thus appear to be a safer medication that could be titrated to a higher dose. However, we noted that this was not the case and Allo was better tolerated than Oxy in the skin models, for reasons that are unclear at the present time. This result led us to test Allo as a potential cutaneous treatment for proliferative skin conditions such as psoriasis.

Topical Allo decreases the incidence of the proliferative cutaneous toxicity from chemotherapy referred to as “hand-foot syndrome” (HFS) [11, 12]. A small phase 2 clinical trial of topical 3% Allo alone against placebo in preventing HFS completed accrual in 2012 and was last modified in 2014. The clinical trial apparently did not meet the trial efficacy in preventing chemotherapy-induced HFS as it has still not been published. This disappointing result supports the rationale of increasing topical agent potency by the addition of another active ingredient to Allo.

To improve the permeation of tissues by Allo, we considered the addition of glutathione (GSH) to disrupt cysteine crosslinks in the epidermis. GSH's physiological relevance is widespread, not only as a potent antioxidant and thiol source but as a regulator of a number of cellular functions, as a detoxifier of endogenous and exogenous toxins, as a key modulator of cellular signaling and proliferation, and as a vital aspect of mitochondrial function. GSH has been previously reported and used as a topical skin whitening agent without ill effects [13] and has been administered safely to people by intravenous injection [14] and by nasal application [15].

## 2. Materials and Methods

There are species differences in enzyme expression. To illustrate, the expression of the crucial enzyme in this study, HGPRT, differs by a factor of 10 between mice and humans [16]. Two cell culture systems for exploring treatments of psoriasis were employed. First, a well-known in vitro model of psoriasis was examined to determine the effectiveness of Allo on cell proliferation and inflammatory signaling. HaCaT keratinocytes were stimulated with the mixture of IL-17A, IL-22, oncostatin M, IL-1alpha, and TNF-alpha (M5) to establish a psoriatic keratinocyte model in vitro [17, 18, 19]. Cell replication and cell survival were analyzed. Then, a three-dimensional reconstructed human psoriatic skin equivalent (PSE) model was used, consisting of normal skin epithelial equivalents (NSEs) established on fibroblast-contracted collagen gels with respective psoriatic dermal cells [20] treated with both Allo and GSH. Examination of cell replication and gene expression, as well as histological analysis of the PSE cells, was performed. The PSE model has been widely utilized in the screening of antipsoriasis treatments [21, 22, 23, 24, 25, 26, 27].

### 2.1. Chemicals and Reagents

The HaCaT cell line was purchased from AddexBio (San Diego, CA). The human psoriatic skin equivalent (PSE) culture system (SOR-300-FT) was from MatTek Corporation (Ashland, MA). Recombinant murine IL-17A, IL-22, IL-1*α*, TNF-*α*, and recombinant human oncostatin M (227 a.a.) were purchased from PeproTech (Cranbury, NJ). All cell culture media and additives were purchased from Thermo Fisher (Waltham, MA) unless otherwise noted. Oxypurinol (Oxy), allopurinol (Allo), and glutathione (GSH) were from Sigma-Aldrich (St. Louis, MO). Other chemicals and reagents were of the highest grade readily available.

### 2.2. Cell Culture

The HaCaT cell line was maintained in Dulbecco's Modified Eagle's Medium (DMEM) supplemented with 10% fetal bovine serum (FBS) and antibiotics (100 U/mL penicillin and 100 mg/mL streptomycin) at 37°C in a humidified 5% CO_2_ incubator. The psoriasis-like keratinocyte model using the M5 cocktail (IL-17A, IL-22, oncostatin M, IL-1*α*, and TNF-*α*, each at a final concentration of 2.5 ng/mL) into the medium of HaCaT keratinocytes has been described previously [19]. Dosing solutions of Oxy and Allo were prepared as discussed previously [28]. HaCaT cells were seeded at approximately 50% density (1 × 10^4^ cells/well [29, 30]), and after 16 h, the medium was replaced with 50 *μ*L of pretreatment medium containing different concentrations of Oxy, Allo (0, 0.5, or 3 mM), MTX (1 *μ*M), or vehicle control and placed back into the incubator. Treatment medium was prepared using standard media containing vehicle control (PBS) or M5 cocktail.

Metabolically active PSE tissues were shipped at 4°C, covered with agarose gel on inserts, one per well of 24-well, medium-supplemented tissue culture plates. Upon receipt, the psoriatic equivalent was equilibrated at 37°C and 5% CO_2_ for 24 h and maintained in SOR-300-FT-MM media. Throughout the experiment, the tissue cultures were maintained at 37°C in a humidified atmosphere containing 5% CO_2_. After every alternate day, fresh prewarmed medium supplemented with vehicle control, Allo (0.5 or 3 mM), GSH (3 mM), a combination of Allo and GSH (3 mM each), or VitD (100 nM) was replenished for 2–6 days. At harvest, each insert was obtained and processed for hematoxylin and eosin staining (H&E), immunostaining, and morphometry. Identically treated plates were sectioned using a sterile razor and processed for RNA extraction as outlined below.

### 2.3. Cytotoxicity Assay

The number of live and dead cells was determined by the MultiTox-Glo Multiplex Cytotoxicity Assay (Promega, Madison, WI) according to the manufacturer's protocol. A dilution series of cells was used to generate a standard curve for calculation of cell number in each well.

### 2.4. Gene Expression

Total RNA was isolated by utilizing the PureLink™ RNA Mini Kit (Invitrogen) according to the manufacturer's instructions. The total RNA was reverse transcribed using the ABI High-Capacity cDNA reverse transcription kit (Applied Biosystems, Foster City, CA). Quantitative real-time polymerase chain reaction (qPCR) was performed with the use of the SYBR Green PCR Master Mix (Applied Biosystems) according to the manufacturer's protocol and amplified on the StepOnePlus Real-Time PCR System (Applied Biosystems).

### 2.5. Pathology/Histology

A total of 11 samples were sent in 10% Neutral Buffered Formalin for grossing, tissue processing, embedding, microtomy, and hematoxylin and eosin staining of the samples. Microtomy sections were cut at 4 microns, and two sections were submitted on each slide, stained with H&E. The slides once stained were cover-slipped and given to the pathologist for interpretation.

## 3. Results and Discussion

**Figure 1:**
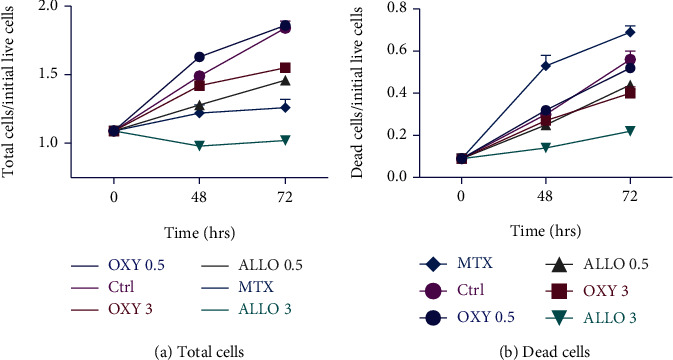
Effects of allopurinol on cell number and viability in the M5-HaCaT model of psoriasis. Cells were treated with allopurinol (Allo, 0.5 or 3 mM), oxypurinol (Oxy, 0.5 or 3 mM), methotrexate (MTX, 1 *μ*M), or vehicle control (0.1 N NaOH) for 48 or 72 hours. Total (live plus dead/input) cell numbers and dead (dead/input) were calculated. Values represent mean ± SEM (*n* = 4).

The HaCaT keratinocyte cell culture model has been shown to elicit many features of the psoriatic phenotype [19]. The analysis involved determining the fraction of total cells at 72 hours compared to the number of live cells at the beginning (time 0), as illustrated in Figure 1(a) and Table 1. The treatment resulted in varying increases in the fraction of total cells: the smallest increase of 3 mM Allo (1.02), followed by MTX, 0.5 mM Allo, and 3 mM Oxy. The highest fraction of total cells was observed with 0.5 mM Oxy (1.9). The maximum fraction of total cells was observed with 0.5 mM Oxy (1.9).

The fraction of dead cells at 72 hours of the total cells at time 0 is shown Figure 1(b). The fraction of dead cells was least affected by 3 mM Allo (0.22) whereas it was most affected by MTX (0.69) followed by 0.5 mM Oxy, 0.5 mM Allo, and 3 mM Oxy. The incremental change in cell death fraction in Table 1 for treatment with Allo 3 mM between day 2 and day 4 is from 0.14 to 0.22 or 0.08 and is consistent with a cell doubling time of about 12 days.

In summary, the results in Figure 1 for this widely used model of psoriasis show that Allo treatment reduces cell proliferation and preserves cell viability much more effectively than both its xanthine oxidase inhibiting metabolite Oxy and a standard agent in clinical use for psoriasis treatment, MTX. Nevertheless, the inconclusive results of the prior clinical trial results for the use of topical Allo (14, 15) and the slower doubling time *in vitro* of single agent Allo of ~12 days in contrast to 30 days in vivo suggest a possible benefit of adding an additional agent to Allo.

To expand on these monolayer culture results with Allo treatment, we interrogated a human psoriatic skin equivalent (PSE) model. We added GSH as a treatment in this 3D tissue culture system. The human PSE culture system is composed of a dermis of psoriatic origin covered by a normal epidermis with an overlying acellular keratin layer (stratum corneum). Psoriasis is marked by an increase in thickness of all three layers.

**Figure 2:**
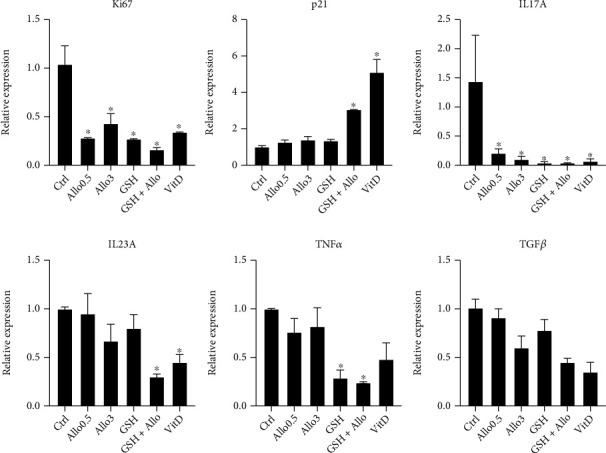
Allopurinol altered gene expression is PSE cells, 2 days post treatment. Data is expressed relative to the control-treated cells (*n* = 3, mean ± SEM). Bars with asterisks are significantly different than the control-treated cells (*p* < 0.05, Dunnett's multicomparison test).

**Figure 3:**
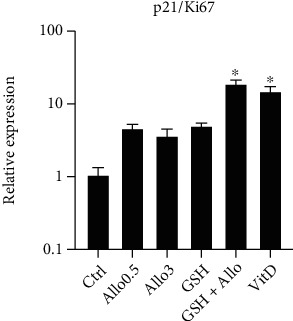
Allopurinol altered markers of cell replication in PSE cells after 2 days of treatment. Data is expressed relative to the control treated cells (*n* = 3, mean ± SEM). Bars with asterisks are significantly different than the control-treated cells (*p* < 0.05, Dunnett's multicomparison test).

The PSE cultures were exposed for up to 6 days to 3.0 mM Allo, 3.0 mM GSH, and 3.0 mM Allo plus 3.0 mM GSH. Vehicle and positive (100 nM vitamin D) control-treated cells were included. Markers of cell proliferation (Ki67), inhibition of cell proliferation (p21), and inflammatory signaling (IL17A, IL23A, TNF*α*, and TGF*β*) were examined by quantitative PCR. As shown in Figure 2 and Table 2, after 2 days of treatment, the PSE cells with Allo or GSH individually significantly decreased Ki67, IL17A, IL23A, and TNF*α* mRNA while increasing the expression of cell replication inhibitory gene p21. To highlight the effects on cell proliferation, the ratio of p21 to Ki67 mRNA levels was examined (Figure 3). After two days of treatment, either Allo or GSH alone or in combination, as well as vitamin D in parallel treatment, demonstrated a markedly increased p21/Ki67 ratio, indicative of decreased cell replication. In all cases, application of Allo and GSH together was a more potent anti-inflammatory/antiproliferative agent than the application of either agent alone.

**Figure 4:**
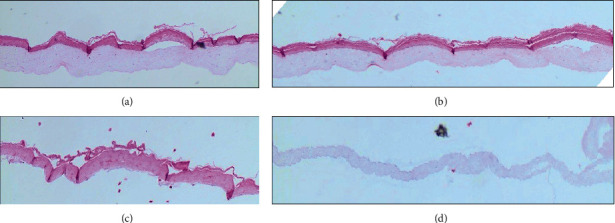
H&E stained PSE cells treated for 6 days with 3 mM allopurinol (a), 3 mM allopurinol plus 3 mM GSH (b), epidermal layer vitamin D (c), or dermal layer vitamin D (d). All slides were photographed with the same magnification (100x). (c) and (d) show markedly increased cell debris after vitamin D treatment.

The H&E staining for cells treated for 6 days with 3 mM Allo and 3 mM Allo plus 3.0 mM GSH is shown in Figures 4(a) and 4(b), respectively. The dermis is the lighter pink layer while the epidermis is the dark pink. The thickness of each of the three tissue layers of skin was monitored and measured by the pathologist (HK) after 2 days and after 6 days of exposure to the treatment medium in a blinded fashion (Table 3).

**Figure 5:**
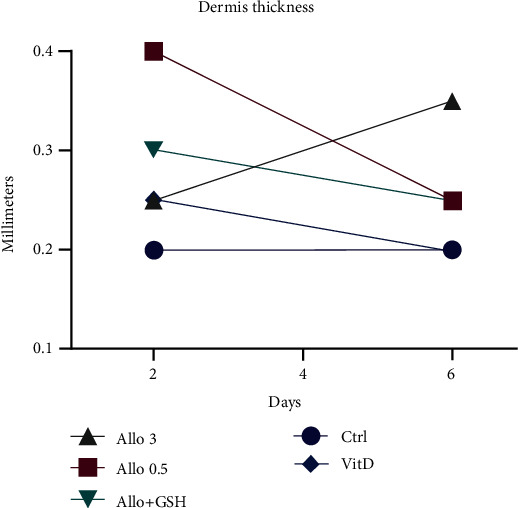
Dermis thickness after treating with allopurinol in PSE cells.

Increased dermis thickness is a feature of psoriasis [31] that is potentially the result of inflammatory signaling or increased cell proliferation from more basal layers of cells. As shown in Table 3 and Figure 5, the combination of Allo and GSH had favorable effects on dermal thickness without signs of toxicity. Raising Allo exposure from 0.5 mM to 3 mM increased day 6 dermis layer thickness (1.4-fold increase day 2 to 6). The treatment with a combination of Allo 3 and GSH decreased the thickness of the dermis (20% decrease day 2 to 6) with a more normal tissue architecture. Also, the tissue integrity with the treatment with both Allo and GSH was preserved. Combined treatment with Allo and GSH at 3 mM resulted in no keratinocyte toxicity (dyskeratosis) and decreased cell presence in the stratum corneum (parakeratosis) after 6 days compared with Allo treatment alone and is evidence of the synergism of Allo and GSH combined therapy. Unfortunately, histological analysis of the 3 mM GSH-treated slide at 6 days was not available for processing due to technical error. However, the gene expression data 2 days post treatment presented in Figure 3 suggests that the dermal thickness data 6-day treatment would not be dramatically affected by the GSH treatment alone. The separation of dermis and epidermis from VitD treatment (Figures 4(c) and 4(d)) may reflect the known toxicity of VitD therapy. Cell debris is noted in the background of Figures 4(c) and 4(d). The decreased dermis width and separation of the dermis from the epidermal layer with vitamin D therapy may reflect toxicity.

Use of Allo and GSH at 3% topically twice a day for 7 weeks resulted in the disappearance of a 3 cm area of psoriatic plaque present for 2 years (J. Ford, personal communication). Additional studies are needed to confirm the benefits of topical Allo and GSH in a human clinical trial.

## 4. Conclusions

The application of Allo in the HaCaT human cell monolayer *in vitro* model of psoriasis shows an unexpected inhibition of cell replication with cell survival significantly improved compared with a standard agent in the treatment of psoriasis, methotrexate. In the MatTek tissue model of psoriasis, the application of GSH alone resulted in strong inhibition of the expression both of the cell replication marker Ki67 and the inflammatory markers IL17A, IL23, TNF alpha, and TNF beta. GSH induced the expression of the replication inhibitor p21. For all anti-inflammatory/antiproliferative markers, the combined application of Allo and GSH was more active than either agent applied separately. With Allo, GSH exerted much less organ culture toxicity than the standard psoriatic agent vitamin D. The combination of Allo and GSH may warrant further study for the treatment of inflammatory skin conditions.

## Figures and Tables

**(a):** 

Time (hrs)	Treatment	Total cell count		
		Live	Dead	Total
0	Ctrl	9177 ± 90^B^^∗^	801 ± 19^G^	9979 ± 73^EF^
48	Ctrl	12393 ± 89^A^	3033 ± 35^DE^	15426 ± 100^B^
48	ALLO 0.5	9386 ± 58^B^	2319 ± 115^EF^	11705 ± 131^D^
48	ALLO 3	7763 ± 51^C^	1275 ± 96^FG^	9038 ± 144^F^
48	MTX	6368 ± 80^D^	4826 ± 379^BC^	11194 ± 445^DE^
72	Ctrl	12199 ± 209^A^	5099 ± 383^B^	17298 ± 444^A^
72	ALLO 0.5	9404 ± 237^B^	4024 ± 166^CD^	13428 ± 216^C^
72	ALLO 3	7363 ± 210^C^	2004 ± 168^EF^	9367 ± 280^F^
72	MTX	5287 ± 306^E^	6300 ± 289^A^	11587 ± 593^D^

**(b):** 

Time (hrs)	Treatment	Relative to initial live cells	
		Total	Dead
0	Ctrl	1.09 ± 0.01^EF^	0.09 ± 0.002^G^
48	Ctrl	1.68 ± 0.01^B^	0.33 ± 0.002^DE^
48	ALLO 0.5	1.28 ± 0.01^D^	0.25 ± 0.012^EF^
48	ALLO 3	0.98 ± 0.02^F^	0.14 ± 0.01^FG^
48	MTX	1.22 ± 0.05^DE^	0.52 ± 0.041^BC^
72	Ctrl	1.88 ± 0.05^A^	0.55 ± 0.042^B^
72	ALLO 0.5	1.46 ± 0.02^C^	0.43 ± 0.018^CD^
72	ALLO 3	1.02 ± 0.03^F^	0.22 ± 0.018^EF^
72	MTX	1.26 ± 0.06^D^	0.69 ± 0.032^A^

^∗^Values are mean ± SEM (*n* = 4). Means with different letters are significantly different (*p* < 0.05, Tukey's multicomparison test).

**Table 2:** Allopurinol altered gene expression is PSE cells, 2 days post treatment.

Trt	Ki67	p21	IL17A	IL23A	TNF*α*	TGF*β*
Control	1.03 ± 0.19	1.00 ± 0.06	1.44 ± 0.79	1.00 ± 0.02	1.00 ± 0.01	1.00 ± 0.08
0.5 mM allopurinol	0.27 ± 0.01	1.27 ± 0.10	0.21 ± 0.07	0.95 ± 0.20	0.75 ± 0.13	0.90 ± 0.09
3 mM allopurinol	0.42 ± 0.09	1.40 ± 0.17	0.09 ± 0.04	0.66 ± 0.17	0.82 ± 0.19	0.6 ± 0.12
3 mM glutathione	0.27 ± 0.01	1.35 ± 0.06	0.04 ± 0.02	0.79 ± 0.14	0.29 ± 0.07	0.77 ± 0.10
3 mM A+3 mM GSH	0.16 ± 0.02	3.06 ± 0.03	0.03 ± 0.01	0.29 ± 0.03	0.23 ± 0.01	0.45 ± 0.04
100 nM VitD	0.34 ± 0.01	5.10 ± 0.70	0.06 ± 0.03	0.45 ± 0.07	0.48 ± 0.17	0.35 ± 0.09

**Table 3:** PSE tissue thickness.

Layer	Time	Layer thickness (mm)					
Treatment		Ctrl	0.5 mM A	3 mM A	3 mM G	3 mM A+G	100 nM VitD
Stratum corneum	2 days	0.02	0.05	0.02	0.15	0.05	0.05
	6 days	0.15	0.05	0.1	N/D	0.1	0.2

Epidermis	2 days	0.1	0.1	0.1	0.2	0.1	0.15
	6 days	0.2	0.1	0.15	N/D	0.15	0.2

Dermis	2 days	0.2	0.4	0.25	0.3	0.3	0.25
	6 days	0.2	0.25	0.35	N/D	0.25	0.2

## Data Availability

Please contact Dr. John Ford (jpfordmd@hotmail.com) for additional information.
